# Shared diagnostic biomarkers and underlying mechanisms between endometriosis and recurrent implantation failure

**DOI:** 10.3389/fendo.2025.1490746

**Published:** 2025-02-19

**Authors:** Hui Li, Chenxu Zhu, Yingjie Gu, Xiaojiao Wei, Xiaowen Wang, Xiaojun Yang, Hong Zhang

**Affiliations:** ^1^ Department of Obstetrics and Gynecology, The First Affiliated Hospital of Soochow University, Suzhou, Jiangsu, China; ^2^ Department of Spinal Surgery, The Third Affiliated Hospital of Soochow University, Changzhou, Jiangsu, China

**Keywords:** endometriosis, recurrent implantation failure, integrated transcriptomic analysis, machine learning, extracellular matrix

## Abstract

**Background:**

Endometriosis (EMs) is a common condition that causes dysmenorrhea, chronic pelvic pain, and infertility, affecting millions of women worldwide. Despite the use of assisted reproductive technology, EMs patients often experience lower embryo implantation rates and recurrent implantation failure (RIF) due to impaired uterine endometrial receptivity. This study aims to identify shared diagnostic genes and underlying mechanisms between EMs and RIF using integrated transcriptomic analysis and machine learning with Gene Expression Omnibus (GEO) datasets.

**Methods:**

We analyzed GSE11691, GSE7305, GSE111974, and GSE103465 as training datasets for EMs and RIF, and GSE25628 and GSE92324 as validation datasets. Differentially expressed genes (DEGs) and Weighted Gene Co-Expression Network Analysis (WGCNA) identified key genes specific to and shared by EMs and RIF. Machine learning algorithms were used to determine the shared diagnostic gene, whose performance was validated in both training and validation datasets. Single-gene Gene Set Enrichment Analysis (GSEA) revealed shared biological processes in EMs and RIF, while CIBERSORT analysis highlighted similarities and differences in immune infiltration between the two conditions. Finally, endometrial samples from healthy controls, EMs, and RIF patients were collected, and qRT-PCR was performed to validate the diagnostic gene.

**Results:**

We identified 48 shared key genes between EMs and RIF. The diagnostic gene EHF was selected through machine learning algorithms, and its diagnostic performance was validated in both training and validation datasets. ROC curve analysis demonstrated excellent diagnostic accuracy of EHF for both diseases. Gene Set Enrichment Analysis (GSEA) revealed that both conditions shared biological processes, including dysregulated extracellular matrix remodeling and abnormal immune infiltration. Furthermore, we validated the expression of EHF in endometrial samples from healthy controls, EMs, and RIF patients. Additionally, we characterized the immune microenvironment in EMs and RIF, highlighting changes in immune cell components associated with EHF.

**Discussion:**

The diagnostic gene EHF identified in this study may serve as a key link between EMs and RIF. The shared pathological processes in both conditions involve alterations in the extracellular matrix and subsequent changes in the immune microenvironment. These findings provide novel insights into potential therapeutic strategies for improving infertility treatment in patients with EMs.

## Introduction

As a complex and enigmatic gynecological disease, endometriosis (EMs) brings dysmenorrhea, chronic pelvic pain, especially infertility to women, which troubles millions of women worldwide (1). Among them, about 30%-50% of patients with EMs are complicated by infertility (2, 3). For women with combined infertility and EMs, Assisted Reproductive Technology (ART) appears to be a promising option (4, 5). It can retrieve oocytes directly from the ovary, avoiding the oocytes from staying in the abdominal microenvironment of EMs, thereby reducing the impact of EMs on the oocytes. However, even when undergoing ART, EMs patients may still experience lower embryo implantation rates or clinical pregnancy rates compared to infertile patients with other causes like tubal blockages (6), and in some cases, even recurrent implantation failure (RIF). These findings suggest the presence of shared pathological processes between these two conditions. Identifying these pathological processes can significantly enhance the pregnancy rates of individuals with EMs.

Meanwhile, the mutation allele frequency (MAF) of cancer-associated genes in endometriotic epithelium significantly increases compared to normal endometrium, indicating a high degree of heterogeneity (7). Endometrial cells carrying cancer-related mutations have a selective advantage in retrograde blood flow, thereby promoting the development of EMs. Therefore, we believe that endometrium in EMs patients is not universally abnormal. Ectopic endometrium seems to more accurately represent the pathological process of EMs.

Recurrent Implantation Failure (RIF) typically refers to the inability of women under the age of 40 to achieve a viable pregnancy after at least three cycles of fresh or frozen embryo transfer, in which more than four high-quality embryos or two high-quality blastocysts have been implanted (8). Current research suggests that the main causes for RIF include embryo developmental defects, uterine disorders, and reduced endometrial receptivity (6, 9, 10). Following ART, local immune dysfunction in the endometrium remains one of the major challenges in RIF, since specific immune activation at the maternal-fetal interface is necessary for embryo invasion (11, 12). Therefore, immune cells in the endometrium, such as natural killer cells, macrophages, and T cells, play a crucial role in regulating endometrial receptivity and embryo implantation (13). However, the mechanisms of immune cell infiltration in patients with RIF still deserves further exploration.

In this study, we aimed to explore the potential diagnostic genes and disease processes shared between EMs and RIF, as well as the changes in the immune microenvironment of these two conditions. To achieve this objective, we downloaded transcriptome data from GEO and identified common hub genes in both diseases through differentially expressed genes (DEGs) analysis and Weighted Gene Co-Expression Network Analysis (WGCNA) respectively. Furthermore, we employed two machine learning algorithms to identify shared diagnostic genes, with EHF being the key gene, and validated its diagnostic performance in the validation dataset. Finally, the single-gene gene set enrichment analysis (GSEA) and immune infiltration analysis showed that both diseases exhibit abnormal extracellular matrix regulation and associated abnormal immune infiltration. In summary, this study provides a theoretical basis for the potential co-pathogenesis of EMs and RIF and provides a new therapeutic direction for the treatment of infertility in EMs patients.

## Materials and methods

### Data collection and preparation

A Total of 6 Gene Expression Omnibus Series (GSE) Datasets retrieved from the Gene Expression Omnibus database (GEO) (http://www.ncbi.nlm.nih.gov/geo/) are included in the Study. For EMs, we have chosen normal endometrial samples and ectopic endometrial samples from EMs patients as our study subjects. Specifically, GSE11691, GSE7305, and GSE25628 were selected, with GSE11691 and GSE7305 serving as the training set, and GSE25628 as the validation set. In the case of the GSE11691 dataset, Principal Component Analysis (PCA) results showed GSM296885 as an outlier (**Supplementary Figure S1**). Consequently, this data has been excluded from subsequent analyses.

**Figure 1:**
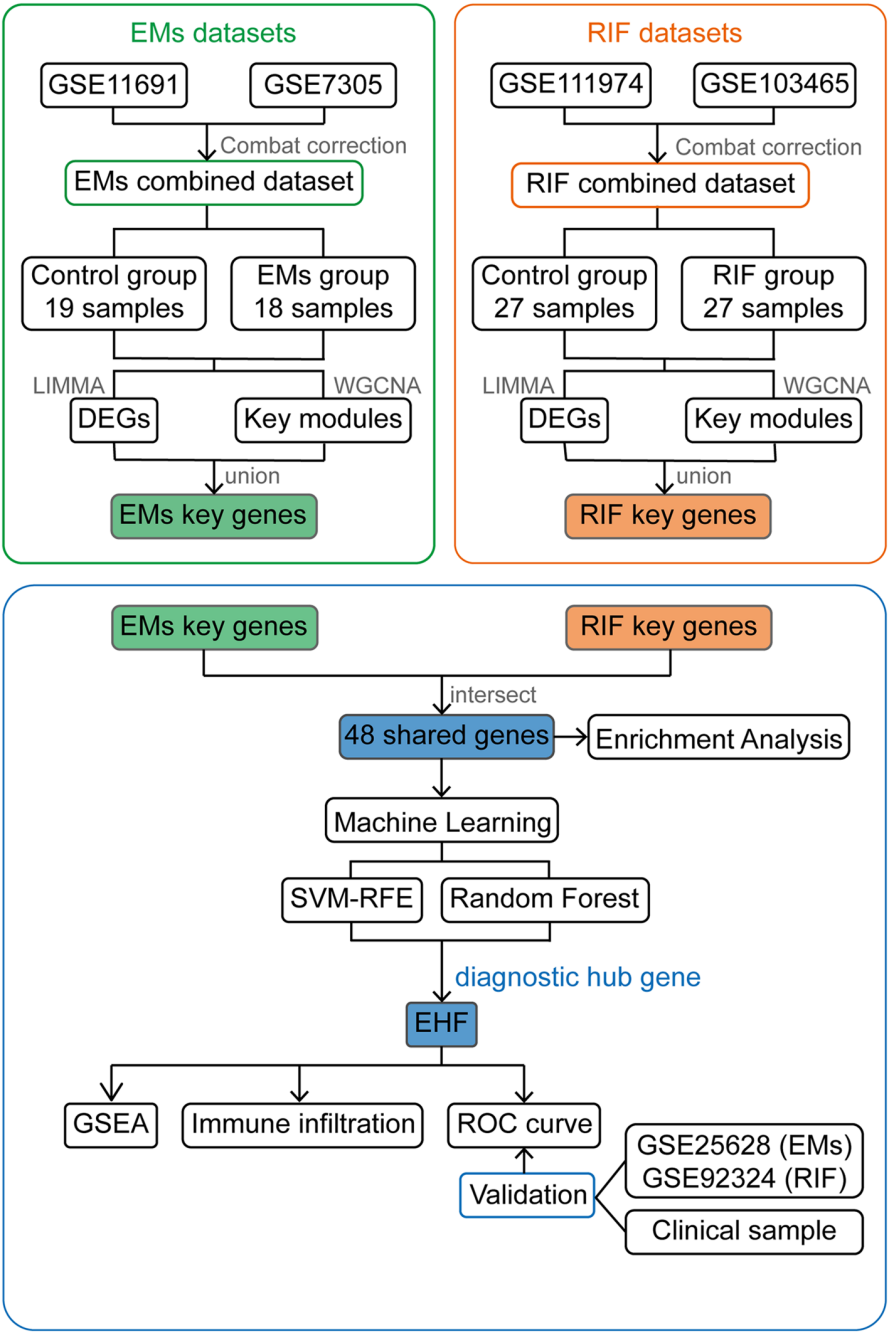
Work flow chart of the entire design. EMs, endometriosis; RIF, Recurrent Implantation Failure; GSE, Gene Expression Omnibus Series; LIMMA, Linear Models for Microarray Data; DEGs, Differentially Expressed Genes; WGCNA, Weighted Gene Co-expression Network Analysis; SVM-RFE, Support Vector Machine- Recursive Feature Elimination; GSEA, Gene Set Enrichment Analysis; ROC curve, The Receiver Operating Characteristic (ROC) curve.

Additionally, GSE111974, GSE103465, and GSE92324 represented RIF data, with GSE111974 and GSE103465 serving as the training set and GSE92324 as the validation set. Detailed information of the datasets was provided in **Table 1** and the whole workflow was shown in **Figure 1**.

**Table 1:** Datasets information.

GSE number	Platform	Samples	Disease	Group
GSE11691	GPL96	8 patients and 9 controls	Endometriosis	Discovery
GSE7305	GPL570	10 patients and 10 controls	Endometriosis	Discovery
GSE25628	GPL571	9 patients and 6 controls	Endometriosis	Validation
GSE111974	GPL17077	24 patients and 24 controls	RIF	Discovery
GSE103465	GPL16043	3 patients and 3 controls	RIF	Discovery
GSE92324	GPL10558	10 patients and 8 controls	RIF	Validation

The “limma” R package was utilized for background correction and normalization in each dataset to ensure uniformity in data processing. After merging the data, a PCA was conducted to assess whether batch effects were present among the datasets. The “sva” package was then employed to correct for batch effects introduced by the different datasets.

### Identification of DEGs

We utilized the “limma” R package to identify DEGs in control and disease groups for the two diseases. The criteria for DEGs selection were set as *P* < 0.05 and |logFC| > 1. The selected DEGs were visualized using volcano plots and heatmaps created with the “pheatmap” R package and “ggplot2” R package.

### Weighted gene co-expression network analysis

To identify hub gene with potential co-regulatory patterns in the dataset, we employed the “WGCNA” R package to perform hierarchical clustering. First, we clustered the samples and removed the filtered outliers. Following that, the “pickSoftThreshold” function was utilized to calculate the linear correlation between the changes in gene connectivity and the number of genes at different soft-thresholding powers. Here, we set the fit index to 0.85 to obtain the optimal β value for subsequent calculations. Then the “adjacency” function constructed an adjacency matrix, adjacencyExpr, and the “TOMsimilarity” function generated the Topological Overlap Matrix (TOM) based on the gene expression data. Hierarchical clustering was performed using the dissimilarity TOM method. Set the minimum number of genes in the module to 60, and then started dynamic pruning module division. Gene modules were obtained, and similar modules were merged. Finally, a heatmap showing the relationship between traits and modules was plotted, displaying correlation coefficients and *P* values.

The genes in each module were sorted according to the module feature values, and the genes were filtered based on the gene significance (GS) and modular membership (MM) values. In EMs and RIF, we selected genes with |MM|>0.8 and |GS|>0.6 as hub genes.

### Identification of shared gene and GO enrichment analysis

By intersecting the DEGs and WGCNA hub genes of EMs and RIF respectively, the key genes involved in the pathological processes of both diseases were obtained. To further identify the biological processes these genes associated with, we conducted the “clusterProfiler “ R package to perform Gene Ontology (GO) enrichment analysis. The enriched pathways were visualized in a bubble chart, displaying the top 10 enriched pathways.

### Machine learning

Two machine learning methods, Support Vector Machine Recursive Feature Elimination (SVM-RFE) and Random Forest (RF), were used to further screen the shared genes between the two diseases.

Firstly, we employed the “RandomForest” R package to identify important genes using the RF algorithm. We constructed a RF model with 500 trees on the training dataset and determined the optimal number of trees through cross-validation error. Finally, genes were ranked according to their importance, and the top 30 most important genes were plotted.

Next, SVM-RFE was employed for further gene selection. Recursive Feature Elimination (RFE) is a backward selection method that starts with all features and recursively removes the least important ones based on the performance of the model. Using the “e1071,” “kernlab,” and “caret” R packages, all 48 genes were initially included in the model, and the optimal number of genes to include in the model was determined through ten-fold cross-validation. SVM-RFE removes one feature each time and calculates Root Mean Square Error (RMSE), which is used to evaluate the error between model predictions and actual observations. The green points in the figure indicated that when the RMSE reaches the minimum value, the corresponding feature subset is considered the best feature set.

### Receiver operating characteristic (ROC) curve

With the “pROC” R package, we generated ROC curves to assess the diagnostic performance of the shared diagnostic genes in both the training and validation datasets. Sensitivity and specificity of these genes were calculated respectively. The sensitivity was plotted on the vertical axis, while specificity was plotted on the horizontal axis. The area under the curve (AUC) was calculated to measure the model performance. A higher AUC value indicates better performance.

### Single-gene gene set enrichment analysis

First, we divided the data sets of the two diseases into EHF high expression and low expression groups according to the median expression of the shared diagnostic gene EHF, and then compared the differences between the two groups and get the logFC of each individual gene. All genes were then ranked based on their logFC values. Subsequently, “clusterProfiler” R package was used to perform GSEA on the sorted genes, with gene sets obtained from the MSigDB database (c5.go.v2023.1.Hs.symbols.gmt). Finally, the top 5 pathways enriched in each group were displayed using the “enrichplot” R package.

### Immune cell abundance analysis

CIBERSORT analysis was performed on each sample in the two diseases. This analysis was based on the principle of linear support vector regression to deconvolve the expression matrix of human immune cell subtypes, so that we can obtain the proportion of immune cells in each sample. LM22 gene expression dataset, which includes data for 22 distinct immune cell types was used to estimate the relative proportions of different immune cell types within complex mixed samples. This dataset can be obtained from the CIBERSORT website (https://cibersort.stanford.edu/).

Following the analysis with the “CIBERSORT” function, we obtained the proportions of 22 immune cell types for each sample. Samples with *P* < 0.05 were used for subsequent analysis. We then used the “corrplot” R package to visualize the proportions of different immune cells in each sample with bar charts. Additionally, the abundance of each immune cell between the normal group and the disease group in the two diseases was calculated, which was displayed by the violin plot of the “vioplot” R package. Finally, the correlation between each immune cell and the shared diagnostic gene EHF was calculated by spearman test, and the results were presented through lollipop charts created with the “ggplot2” R package.

### Human endometrial samples

All endometrial samples were obtained from the First Affiliated Hospital of Soochow University. Ectopic endometrium samples were obtained from EMs patients through laparoscopic procedures. These surgeries were conducted based on clinical indications for the diagnosis and management of endometriosis. The inclusion criteria for this study were as follows: (1) Laparoscopic diagnosis of grade III or IV EMs; (2) Aged 20-40 years old; (3) No hormonal treatment received in the three months preceding sample collection; (4) Exclusion of other uterine or endocrine disorders. RIF samples were collected from secretory phase endometrium of patients who had undergone at least two cycles of IVF, ICSI, or frozen embryo transfers, with a cumulative transfer of at least four high-quality cleavage-stage embryos or two high-quality blastocysts and still failed to implant. PCOS and other uterine or endocrine disorders were excluded. Normal endometrial samples were sourced from healthy women aged 20-40 during the secretory phase of the menstrual cycle.

The collected endometrial samples were processed using Trizol (Vazyme, Nanjing, China) for RNA extraction and then reverse-transcribed into cDNA using HiScript III 1st Strand cDNA Synthesis Kit (Vazyme, Nanjing, China). Subsequently, quantitative real-time polymerase chain reaction (qRT-PCR) was employed to assess the relative expression levels of EHF in each sample. The primer of the genes we used were showed in **Supplementary Table S1**.

The results of the qRT-PCR were presented as ΔΔCT values for each sample. The comparison between the two groups was performed using a t-test, and the results were displayed as the mean ± standard error of the mean (SEM). *P* < 0.05 was considered statistically significant.

## Results

### Data collection and preparation

**Figure 2:**
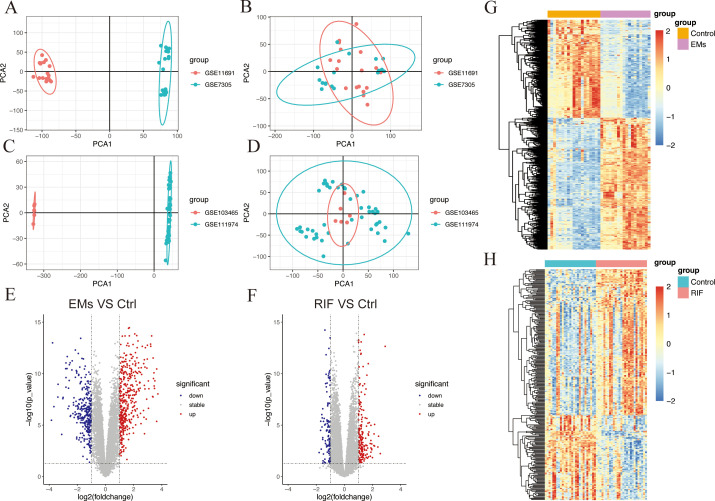
Data preparation and DEGs identification in EMs and RIF. **(A, B)** PCA plots showed the expression pattern before batch correction **(A)** and after batch correction **(B)** in GSE11691 and GSE7305 of EMs group. **(C, D)** PCA plots showed the expression pattern before batch correction **(C)** and after batch correction **(D)** in GSE103465 and GSE111974 of RIF group. **(E, F)** Volcano plot showed the DEGs (*P* < 0.05 and |logFC| > 1) in EMs group **(E)** and RIF group **(F)**. Blue showed the down-regulated genes and red showed the up-regulated genes. **(G, H)** Heatmap showed the DEGs (*P* < 0.05) in EMs group **(G)** and RIF group **(H)**. Blue showed the down-regulated genes and red showed the up-regulated genes.

After performing background correction and normalization on each data set respectively, we merged the data and corrected for batch effects. The PCA results showed the data before and after correcting for batch effects in EMs (**Figures 2A, B**) and RIF (**Figures 2C, D**).

### Identification of differentially expressed genes

The “limma” R package was utilized to analyze DEGs between the ectopic endometrium of EMs patients and the normal group. The criteria for DEGs selection were set as *P*< 0.05 and |logFC| > 1. A total of 708 DEGs were identified, consisting of 406 upregulated genes and 302 downregulated genes. Additionally, there were 304 DEGs in the RIF dataset, of which 187 genes were up-regulated, and 117 genes were down-regulated. The overall profiles of these DEGs were depicted through volcano plots and heatmaps (**Figures 2E–H**), highlighting their significant role in the progression of both EMs and RIF disease.

### Identification of key WGCNA modules

To further identify key genes associated with the disease, WGCNA was employed to identify the most disease-relevant modules in the two groups.

**Figure 3:**
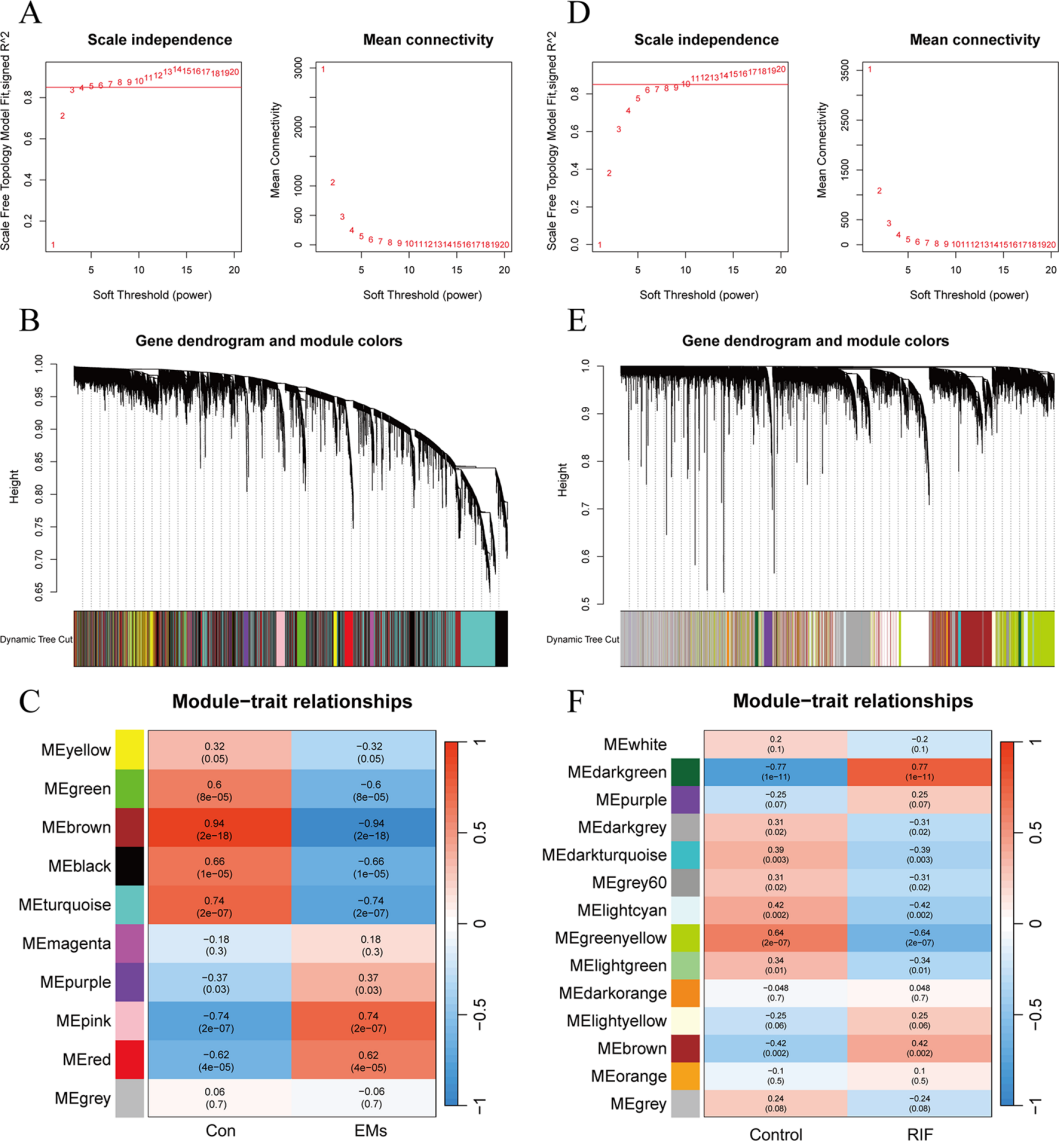
Weighted gene co-expression network analysis of EMs and RIF. **(A)** Determination of soft-threshold power in the WGCNA of EMs group. **(B)** Clustering dendrograms showing modules of highly connected genes within the EMs group. **(C)** Heatmap of the correlation between the modules and traits of EMs group. Red indicates positive correlations, blue represents negative correlations, and the correlation coefficients and P values are displayed within the individual grid cells. **(D)** Determination of soft-threshold power in the WGCNA of RIF group. **(E)** Clustering dendrograms showing modules of highly connected genes within the RIF group. **(F)** Heatmap of the correlation between the modules and traits of RIF group.

In EMs group, an optimal soft threshold of 5 (R^2^ = 0.85) was selected for constructing a scale-free network, based on calculations of scale-independence and average connectivity (**Figure 3A**). Subsequently, an adjacency matrix was generated using the adjacency function. As shown in **Figure 3B**, hierarchical clustering was performed using the TOM dissimilarity measure. The genes were clustered based on their expression correlation and divided into different groups, resulting in a total of 10 gene modules. As shown in **Figure 3C**, with a significance level of *P* < 0.05, genes in the Green, Brown, Black, and Turquoise modules were positively correlated with clinical features of the control group and negatively correlated with the EMs group. However, the Purple, Pink, and Red modules exhibited a negative correlation with clinical features of the control group and a positive correlation with the EMs group.

As for RIF group, the selected optimal soft threshold for power was 10 (R^2^ = 0.85) (**Figure 3D**). After performing hierarchical clustering using the TOM (**Figure 3E**), a total of 14 modules were identified. As shown in **Figure 3F**, with a significance level of *P* < 0.05, genes in the Darkgray, Darkturquoise, Gray60, Ligntcyan, Greenyellow, and Lightgreen modules were positively correlated with clinical features of the control group and negatively correlated with the RIF group. However, the Darkgreen and Brown modules exhibited a negative correlation with clinical features of the control group and a positive correlation with the RIF group.

Among the selected key modules, a total of 709 genes were identified in the EMs group and 290 genes in the RIF group, satisfying the criteria of |MM| > 0.8 and |GS| > 0.6.

### GO enrichment analysis of shared genes

**Figure 4:**
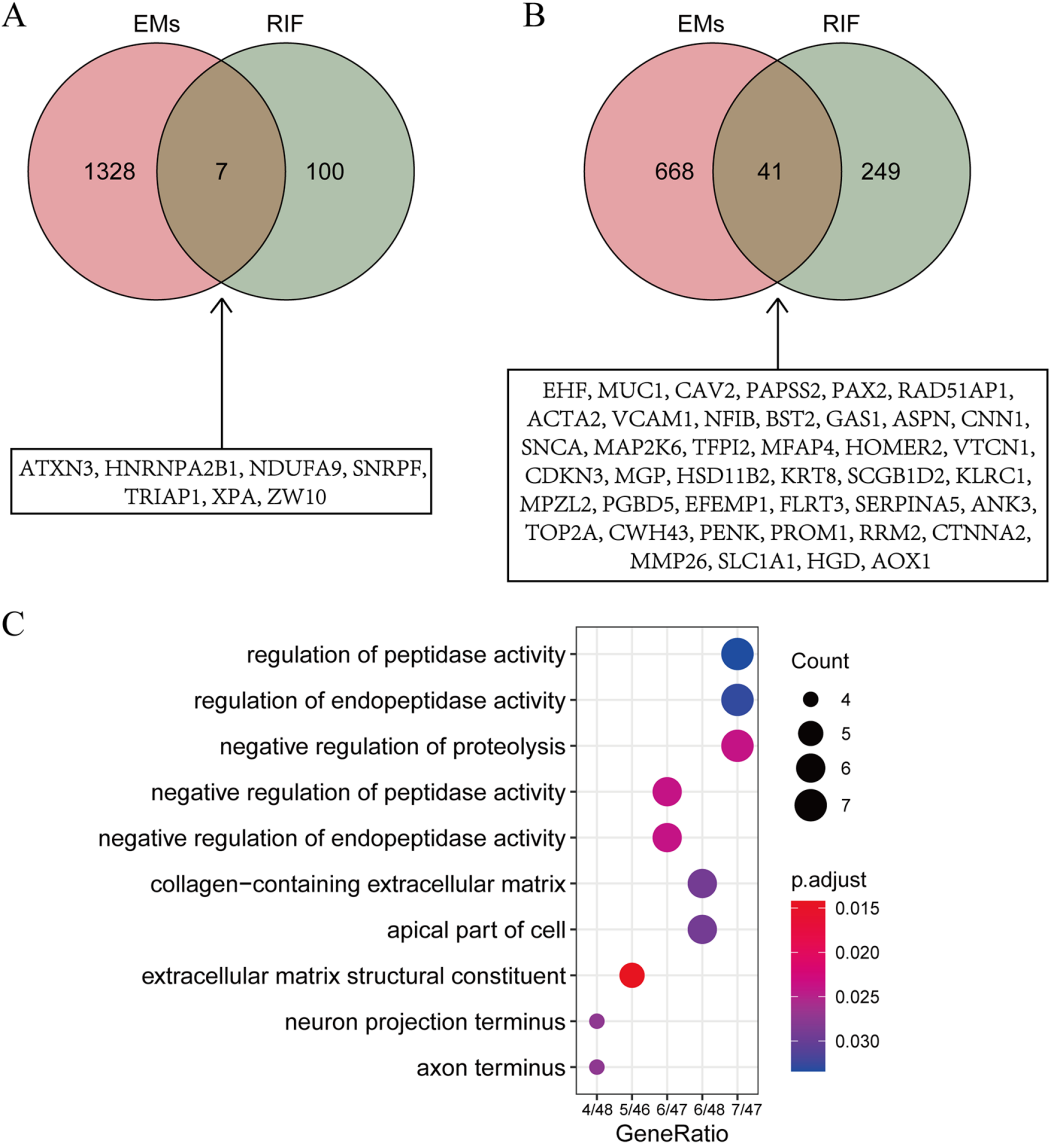
Identification of shared gene functions. **(A)** Intersection of DEGs from EMs and RIF. The specific information of the intersection gene is displayed in the box. **(B)** Intersection of key modules from EMs and RIF. The specific information of the intersection gene is displayed in the box. **(C)** GO function enrichment analysis of shared genes within boxes from panels A and **(B)** The X-axis stands for count of genes. The Y-axis represents the enriched pathways.

To explore the shared pathological mechanisms between EMs and RIF, we have identified overlapping genes from DEGs and key modules obtained through WGCNA. As shown in **Figures 4A** and **B**, it appears that there were 7 genes shared between the DEGs, and 41 genes shared between the key modules identified by WGCNA.

To further investigate the biological processes associated with these genes, we conducted Gene Ontology (GO) enrichment analysis (**Figure 4C**). The results indicated that these genes are primarily enriched in biological processes related to regulation of peptidase activity and extracellular matrix structural constituent, suggesting that alterations in extracellular matrix structural components play a significant role in both EMs and RIF.

### Identification of shared diagnostic gene

To further identify the genes that serve as potential biomarkers for disease diagnosis or classification, we applied RF and SVM-RFE algorithms to each group.

**Figure 5:**
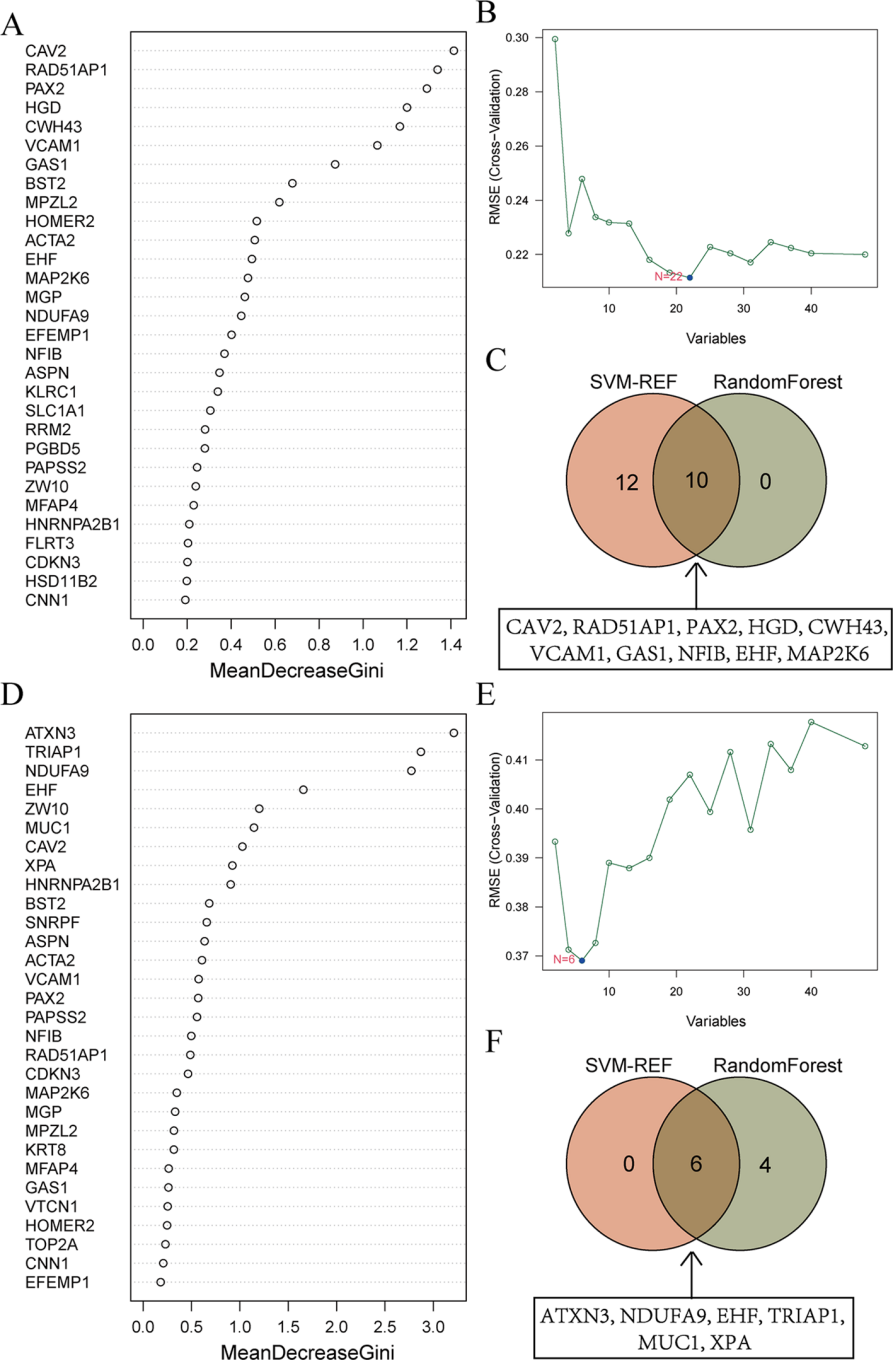
Machine learning screens for shared diagnostic genes. **(A)** Importance ranking of top 30 genes in random forest from EMs. The X-axis stands for the importance score of genes calculated by the Gini coefficient method. The Y-axis represents the names of genes. **(B)** SVM-RFE algorithm screened 22 diagnostic markers in EMs. The X-axis represents the number of genes included in the model. The Y-axis represents the RMSE value calculated each time a gene is deleted. **(C)** Intersection of genes selected from RF and SVM-RFE algorithm in EMs. The specific information of the intersection gene is displayed in the box. **(D)** Importance ranking of top 30 genes in random forest from RIF. **(E)** SVM-RFE algorithm screened 6 diagnostic markers in RIF. **(F)** Intersection of genes selected from RF and SVM-RFE algorithm in RIF.

48 candidate genes were input into the RF algorithm, and the genes were ranked based on their importance scores, as shown in **Figure 5A**. Subsequently, when screening the 48 candidate genes for EMs using SVM-RFE, it was observed that the RMSE was minimized when the number of genes was set to 22 (**Figure 5B**). The top 10 genes in the RF ranking were then intersected with the 22 genes selected by SVM-RFE, resulting in a set of 10 overlapping genes (CAV2, RAD51AP1, PAX2, HGD, CWH43, VCAM1, GAS1, NFIB, EHF, MAP2K6) (**Figure 5C**).

Similarly, for RIF, the RF algorithm results displayed the genes ranked by their importance scores, as depicted in **Figure 5D**. The SVM-RFE algorithm was applied to the 48 candidate genes, resulting in the identification of 6 hub genes (**Figure 5E**). The intersection of the top 10 genes from the RF ranking and the 6 genes selected by SVM-RFE yielded a set of 6 overlapping genes, specifically ATXN3, NDUFA9, EHF, TRIAP1, MUC1, and XPA (**Figure 5F**).

### Validation of shared diagnostic genes in training and validation datasets

**Figure 6:**
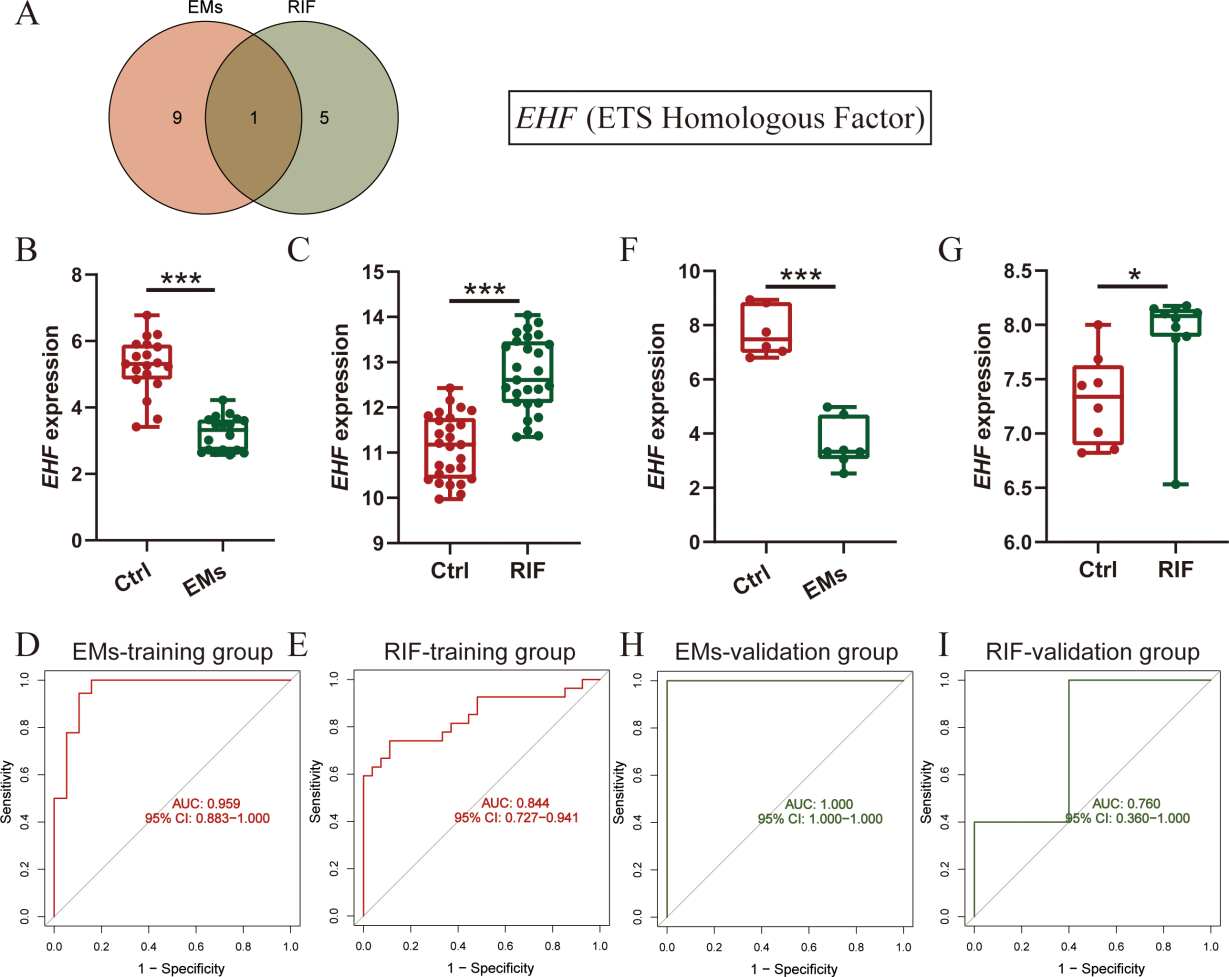
Diagnostic efficacy and verification of shared diagnostic genes. **(A)** Intersection of diagnostic genes selected from EMs and RIF. **(B, C)** EHF expression in EMs **(B)** and RIF **(C)** training group. **(D, E)** ROC curve of EHF in EMs **(D)** and RIF **(E)** training group. The X-axis represents specificity and the Y-axis represents sensitivity. **(F, G)** EHF expression in EMs **(F)** and RIF **(G)** validation group. **(H, I)** ROC curve of EHF in EMs **(H)** and RIF **(I)** validation group. *P < 0.05, ***P < 0.001.

To further understand the shared physiological processes, we took the intersection of the most diagnostically valuable genes obtained from EMs and RIF (**Figure 6A**). We found that the diagnostic gene shared between the two conditions was EHF (Ets homologous factor).

Next, we assessed the expression levels of EHF in both EMs and RIF. The results revealed that in EMs, the expression of EHF was significantly lower than that in control group (*P* < 0.001), while in RIF, the expression of EHF was significantly higher than that in control group (*P* < 0.001) (**Figures 6B, C**). Additionally, to assess the diagnostic sensitivity and specificity of EHF, ROC analysis was performed separately for EMs and RIF. The results indicated that the AUC for EMs and RIF were 0.959 and 0.844, respectively (**Figures 6D, E**).

Moreover, we conducted external validation for the expression levels and diagnostic efficacy of EHF in both diseases. For EMs, we utilized the GSE25628 dataset, where the expression of EHF was significantly lower than in the control group (*P* < 0.001), consistent with the trend observed in the training dataset (**Figure 6F**). Regarding RIF, we employed the GSE92324 dataset, where the expression of EHF was significantly higher than in the control group (*P* < 0.05), aligning with the training dataset’s trend (**Figure 6G**). Subsequently, we also verified the sensitivity and specificity of EHF diagnostic performance in these two validation sets. The results demonstrated AUC values of 1.0 for EMs and 0.760 for RIF (**Figures 6H, I**). These results indicated that EHF is concurrently involved in the development of both EMs and RIF, with excellent diagnostic efficacy in both conditions.

### Single-gene GSEA of EHF

**Figure 7:**
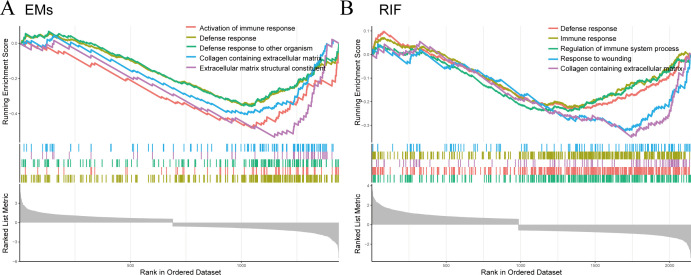
GSEA for EHF in EMs and RIF. **(A)** GSEA enrichment analysis for EHF in EMs. The X-axis represents the sorted genes, and the Y-axis represents the corresponding Running Enrichment Score (ES). **(B)** GSEA enrichment analysis for EHF in RIF.

Subsequently, we performed Single-Gene GSEA enrichment analysis on EHF separately for EMs and RIF (**Figures 7A, B**). The results revealed that the immune response and collagen containing extracellular matrix pathways were activated in both two diseases.

### Immune infiltration analysis of shared diagnostic genes

**Figure 8:**
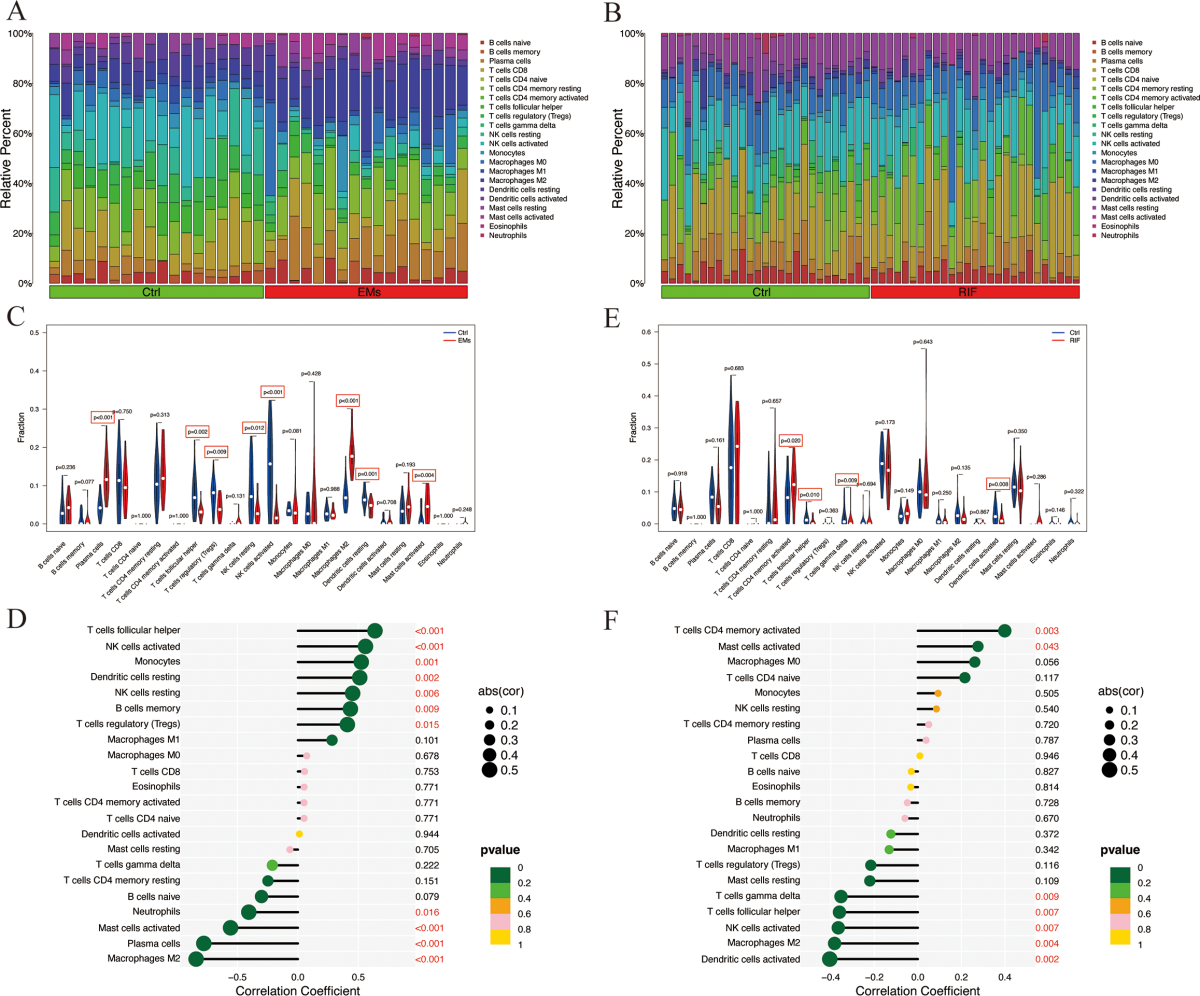
Immune infiltration analysis of EMs and RIF. **(A)** Relative abundance of 22 immune cell types in each sample of EMs. **(B)** Relative abundance of 22 immune cell types in each sample of RIF. **(C)** Relative expression of each immune cell in the control group and EMs group. **(D)** Correlation score between EHF and immune cells in EMs group. **(E)** Relative expression of each immune cell in the control group and RIF group. **(F)** Correlation score between EHF and immune cells in RIF group.

Since both EMs and RIF exhibit activated immune response-related pathways, we conducted an analysis of the abundance of immune cells in each sample using CIBERSORT. **Figures 8A** and **B** displayed the relative abundance of 22 types of immune cells in each sample, revealing significant differences in macrophages, NK cells, and mast cells between EMs and RIF.

Compared to the control group, the EMs group exhibited a significant increase in plasma cells, M2 macrophages, and activated mast cells, while T cell follicular helper, Tregs, NK cells, and dendritic cells showed a significant decrease (**Figure 8C**). Notably, T cells follicular helper, NK cells, monocytes, dendritic cells, B cells memory, and macrophages M1 were significantly positively correlated with EHF expression (*P* < 0.05), whereas macrophages M2, plasma cells, mast cells, and neutrophils showed a significant negative correlation with EHF expression (*P* < 0.05) (**Figure 8D**). In the case of RIF, T cells CD4 memory showed a significant increase compared to the control group, while T cells follicular helper, T cells gamma delta, and dendritic cells exhibited a significant decrease (**Figure 8E**). Notably, T cell CD4 memory and mast cells were significantly positively correlated with EHF expression (*P* < 0.05), whereas dendritic cells, macrophages M2, NK cells, T cells follicular helper, and T cells gamma delta were significantly negatively correlated with EHF expression (*P* < 0.05) (**Figure 8F**). These results indicate that immune cells play a crucial role in the pathogenesis of both EMs and RIF and are significantly associated with EHF.

### Validation of EHF in clinical sample

**Figure 9:**
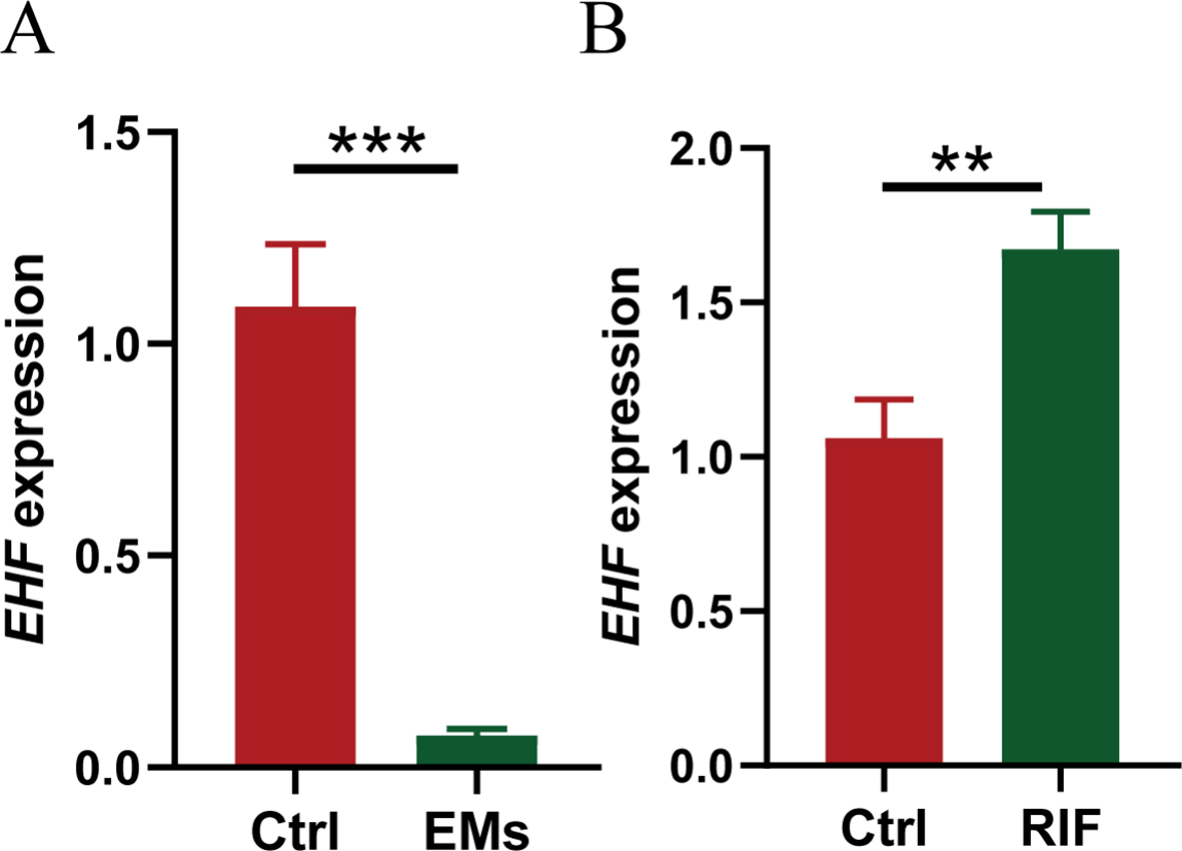
Validation of EHF expression in EMs and RIF. **(A)** The relative mRNA expression levels of EHF in normal (n=3) and EMs patients (n=4). **(B)** The relative mRNA expression levels of EHF in normal (n=3) and RIF patients (n=4). **P < 0.005, ***P < 0.001.

Furthermore, ectopic endometrial samples were obtained from patients with EMs, and secretory-phase endometrial samples were collected from patients with RIF and healthy controls. The expression of EHF in these tissues was analyzed using qRT-PCR, and the results were consistent with the above data analysis. Compared to the control group, EHF expression was significantly decreased in the EMs group and significantly increased in the RIF group (**Figures 9A, B**).

## Discussion

While it is now clear that the primary defects in EMs-related infertility are in the ovaries and oocyte quality, further studies have shown that the uterine endometrial receptivity in EMs may also be compromised (6). Prapas et al. conducted a study in which oocytes from the same donor were implanted into women with and without EMs. By eliminating the confounding effects of EMs on oocyte quality, they found a significant reduction in implantation rates among EMs patients (6). Therefore, it is necessary to explore why the endometrial receptivity of patients with EMs is poor and its similarities and differences with the endometrium of patients with RIF.

As for the cause of EMs, the theory of menstrual reflux proposed by Sampson et al. in 1922 is still recognized by most scholars. Takayuki et al. have demonstrated the heterogeneity of the genomic structure of endometrial epithelium by sequencing single endometrial glands (7). Furthermore, through whole-exome sequencing of ovarian endometriotic and normal uterine endometrial epithelium, they observed a significant increase in the mutant allele frequency (MAF) of cancer-related genes within endometrial ectopic epithelium (7). Their findings suggest that endometrial cells already carrying cancer-associated mutations have a selective advantage in retrograde flow at ectopic sites, thereby contributing to the development of EMs. Inversely, Taylor et al. demonstrated that by implanting GFP-labeled mouse uterine endometrial cells into the abdominal cavity of recipient mice, GFP fluorescence also appeared around the eutopic endometrium blood vessels of the recipient mice (14). This finding provides evidence that some ectopic endometrium may potentially be re-implanted into the uterine cavity through circulation, thus affecting the local microenvironment in the uterus. These studies all suggest the dispersion and mobility of eutopic EMs lesions. Hence, we hypothesize that eutopic endometrium in EMs patients is not universally abnormal. Ectopic endometrium appears to more accurately represent the pathological process of EMs.

It is well known that endometriosis lesions are characterized by significant infiltration of immune cells and secretion of large amounts of extracellular matrix due to the menstrual cycle, implantation of foreign tissues and repeated damage and repair of the endometrium at these sites, eventually leading to fibrosis (15). Also, during the endometrial preparation process for embryo implantation, several key events, such as endometrial decidualization, trophoblast chemotaxis, attachment, migration and invasion processes, all involved the transformation of the extracellular matrix. When any issues arise during these processes, it can substantially impact uterine receptivity, leading to RIF. Consistent with these established viewpoints, our intersection of hub genes in these two diseases revealed significant enrichment related to the regulation of peptidase activity and extracellular matrix component composition, which are closely associated with alterations in extracellular matrix components and collagen accumulation. Furthermore, recent studies have suggested that endometrial scratching may improve implantation rates in RIF patients by promoting endometrial receptivity and enhancing extracellular matrix remodeling (16). This finding aligns with our results, highlighting the potential role of extracellular matrix regulation in RIF.

ETS homologous factor (EHF) is primarily expressed in glandular organs and plays a crucial role in the proliferation and differentiation of epithelial cells (17). Currently, the role of EHF in tumor progression has been relatively well-studied. For example, the loss of EHF has been found to promote epithelial-mesenchymal transition and cell migration in conditions such as prostate cancer (18, 19), lung cancer (20, 21), pancreatic cancer, and esophageal squamous cell carcinoma. On the other hand, in gastric cancer (22), thyroid cancer, and ovarian cancer (23), EHF has been shown to promote cell proliferation. Meanwhile, the role of EHF in normal tissues has also been reported. In a study involving whole-body EHF knockout mice, it was found that the EHF transcription factor plays a crucial role in maintaining the homeostasis of normal epidermal and intestinal epithelial cells (24). However, the role of EHF in endometrial epithelial cells is still a subject for future research. Given the excellent diagnostic efficiency of EHF for EMs and RIF, further exploration of the relationship between EHF and these conditions led us to perform single-gene GSEA for EHF in both diseases. The results showed that the Collagen containing extracellular matrix pathway was downregulated in both diseases. Similarly, Peng Hou et al. (24) observed that overexpression of EHF in MGC803 cells (a kind of gastric cancer cells) led to a significant upregulation of MMP-2, -7, -9, and -14, along with decreased E-cadherin expression and increased vimentin expression. This suggests that EHF may contribute to the remodeling of the extracellular matrix (ECM) through the regulation of MMPs, while also influencing the epithelial-to-mesenchymal transition (EMT) process. However, how EHF regulates the ECM in the endometrium remains to be further investigated. Further research is required to investigate the impact of EHF on EMT in EMs ectopic lesions and its role in decidualization in RIF. We propose that EHF could serve as a biomarker for identifying patients at higher risk of implantation failure and as a potential target for therapeutic interventions aimed at improving uterine receptivity. Moreover, the differences in EHF expression between normal endometrium and the endometrium of patients with endometriosis remain to be further explored.

The immune microenvironment system also plays a role that cannot be ignored in EMs (25). Our results indicate an increase in plasma cells within EMs ectopic lesions, suggesting a heightened autoimmune response at the lesion site, which is related to the high recurrence of EMs. Epidemiological studies also showed that patients with EMs have a higher incidence of other autoimmune diseases (26, 27). Tregs have immunosuppressive properties, typically inhibiting or downregulating the induction and proliferation of effector T cells. Studies have suggested that IL-33 derived from endometriotic stromal cells may induce type 2 immune response by stimulating Treg cells to secrete Th2 cytokines and promote lesion progression and local fibrosis formation (28). Additionally, other studies have shown that the abundance of Treg cells is reduced in endometriotic lesions compared to normal endometrium, which exacerbates local inflammation and angiogenesis, and is similarly associated with the progression of endometriotic lesions (29). This finding is consistent with our results. The immune microenvironment influences various stages of disease onset and progression. Further studies are needed to investigate the role of Treg cells in the development of endometriotic lesions. Previous studies have shown that NK cell activity is decreased in EMs patients (30), primarily manifested by reduced cytotoxicity of NK cells from their peripheral blood and peritoneal fluid toward K562 cells (31–33). The main contributing factor may be an increase in the expression of certain inhibitory NK cell receptors (31, 34). In the present study, we found a decreased abundance of NK cells in endometriotic lesions, which may partially explain the survival of ectopic endometrium colonization and impaired elimination. Alongside the reduction in NK cells, M2 macrophages also play a crucial role in the survival of endometriotic implants (35). Due to the decrease in the M1/M2 ratio, there is insufficient cytotoxicity for eliminating endometriotic lesions and a promotion of angiogenesis (36–38). The aberrant infiltration of these immune cells collectively contributes to the abnormal extracellular matrix in the local lesions of EMs, promoting the fibrotic formation of local lesions. Furthermore, it further affects the intrauterine microenvironment, resulting in decreased implantation and pregnancy rates for EMs patients.

The endometrium undergoes a series of complex changes during the menstrual cycle to prepare for embryo implantation, a process that requires precise coordination between the embryo and the endometrium (39, 40). Immune factors, including various immune cell populations and their intricate signaling pathways, are involved in the establishment of immune tolerance or inflammation at the maternal-fetal interface, which can regulate endometrial receptivity and implantation (41). Previous studies have shown that Treg cells were significantly reduced in both peripheral blood and endometrium of patients with RIF. Human chorionic gonadotropin (hCG) can regulate the differentiation of Tregs, thereby affecting the pregnancy outcome in RIF women (42). Furthermore, dendritic cells and Tregs are closely interconnected and mutually influential (43). In our study, however, no significant difference in Tregs expression was observed between normal endometrium and RIF endometrium, while dendritic cells were significantly reduced. Any alteration in these cells can affect other immune cell populations, ultimately disrupting immune homeostasis and normal embryo implantation. Therefore, phenotypic balance within the immune cell population is critical for establishing endometrial receptivity to implantation (39). From this point of view, the eutopic endometrium of EMs needs further study, which is also our future research direction.

## Conclusions

In conclusion, our study has identified the shared diagnostic gene EHF in EMs and RIF. We have also explored the common pathological changes in these two diseases, which include alterations in the extracellular matrix and the subsequent changes in the immune microenvironment. Our analysis further deepened the understanding of the underlying pathogenic mechanisms shared between EMs and RIF, offering novel therapeutic avenues for addressing infertility in EMs patients.

## Data Availability

The datasets presented in this study can be found in online repositories. The names of the repository/repositories and accession number(s) can be found in the article/**Supplementary Material**.
