# Supplementary material for: Shared diagnostic biomarkers and underlying mechanisms between endometriosis and recurrent implantation failure
Source: Front Endocrinol (Lausanne). 2025 Feb 19;16:1490746. doi: 10.3389/fendo.2025.1490746 (PMC11879817; doi:10.3389/fendo.2025.1490746)
Supplement: Supplementary file 1 [file DataSheet1.docx]

**Supplementary Figure 1**

**
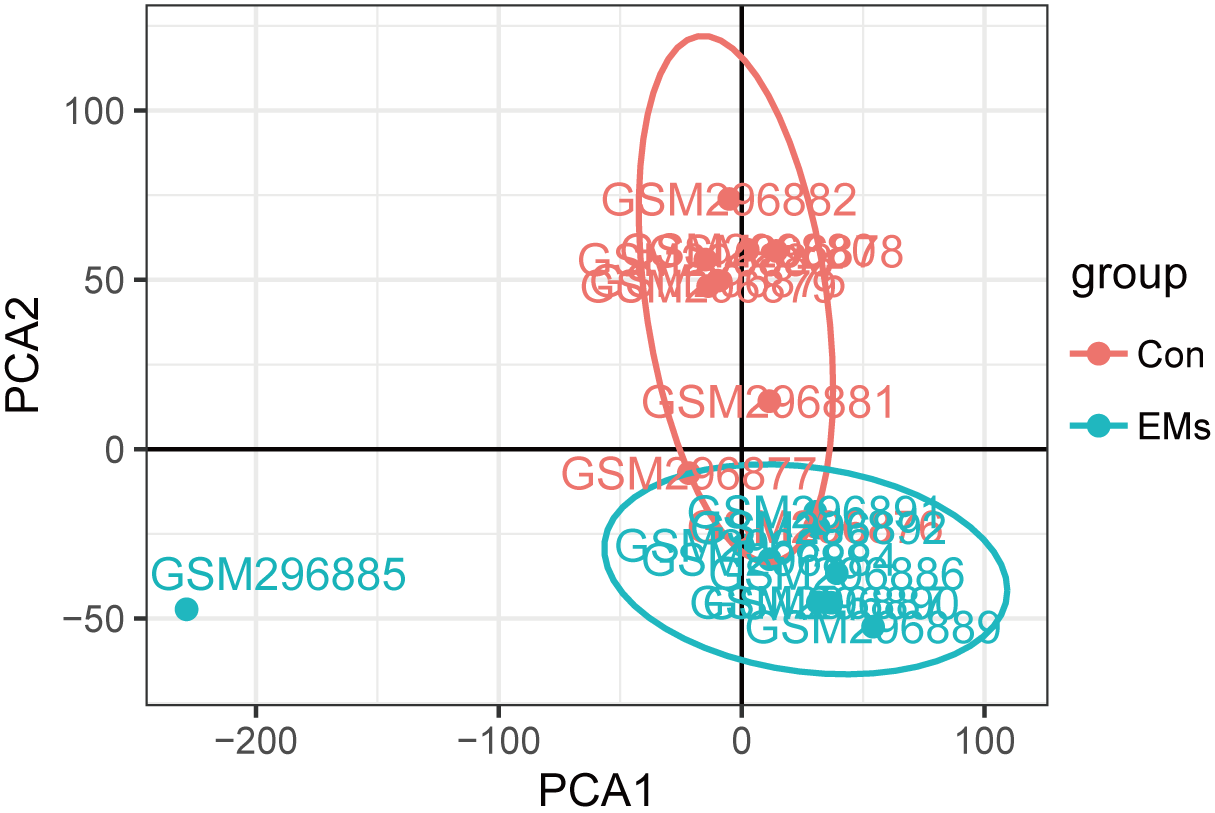
**

**Supplementary Fig1.** PCA plot showed the expression pattern after background correction and normalization in GSE11691 of EMs group. GSM296885 was considered an outlier and will be removed in the following analysis.

**Supplementary Table 1**

Primers of genes used in qRT-PCR analyses

| Gene | Forward | Reverse |
| --- | --- | --- |
| *EHF* | ACCAAGTACCAGGTGTGGGAGTG | GAGGTGCTCGCCGTTGATGTC |
| *Gapdh* | GGAGCGAGATCCCTCCAAAAT | GGCTGTTGTCATACTTCTCATGG |
